# Periconceptional stressors and social support and risk for adverse birth outcomes

**DOI:** 10.1186/s12884-020-03182-6

**Published:** 2020-08-24

**Authors:** Kari A. Weber, Suzan L. Carmichael, Wei Yang, Sarah C. Tinker, Gary M. Shaw, National Birth Defects Prevention Study

**Affiliations:** 1 Division of Neonatal and Developmental Medicine, Department of Pediatrics, Stanford University School of Medicine, 1265 Welch Road x1C21, Stanford, California, 94305 USA; 2 National Center on Birth Defects and Developmental Disabilities, Centers for Disease Control and Prevention, Atlanta, GA USA

**Keywords:** Stress, Support, Preterm, Birthweight, Maternal, Adverse pregnancy outcomes

## Abstract

**Background:**

The prevalence of preterm birth and low birth weight has been increasing slightly in recent years. A few studies have suggested that psychosocial stress during pregnancy may increase risk for these adverse birth outcomes. To extend those observations, we analyzed various major life event stressors separately and cumulatively as potential risk factors for preterm birth and low birth weight using granular categories of each outcome in a large, population-based study. Additionally, we assessed if greater social support buffered any effects.

**Methods:**

Data were from a nested prevalence study of 4395 women in the National Birth Defects Prevention Study who delivered live-born non-malformed infants (controls) between 2006 and 2011. Participants completed a standardized, computer-assisted interview between 6 weeks and 24 months after delivery that included questions on stress and social support from 3 months before pregnancy to the 3rd month of pregnancy. Cumulative stress and support indices were also calculated. Preterm birth was divided into “early preterm” (< 32 weeks), “late preterm” (32–36 weeks) and “term.” Low birthweight was divided into “very low birth weight” (< 1500 g), “low birth weight” (1500–2499 g) and “normal birth weight” (≥2500 g). Relative risks and 95% confidence intervals (95% CI) were calculated using Poisson regression.

**Results:**

For women reporting relationship difficulties, there was a suggestive risk of early preterm birth (RR: 1.9, 95%CI: 0.9–3.9) and very low birthweight (RR: 2.0, 95%CI: 0.9–4.4). For women reporting that they or someone close to them were victims of abuse, violence, or crime, there was an increased risk of low birthweight (RR: 1.8, 95%CI: 1.1–2.7) and late preterm birth (RR: 1.5, 95%CI: 1.0–2.2). There were no strong associations observed between social support questions and the various outcomes.

**Conclusions:**

Our results add some support to prior evidence that certain stressors may be associated with increase selected adverse birth outcomes risk. We did not find strong evidence that social support buffered the observed risks in our study.

## Background

Roughly 10% of births in the United States are delivered preterm (before 37 weeks of gestation), and 8% of infants are born at low birthweight (< 2500 g); the prevalence of both has been increasing slightly in recent years [1]. One potential risk factor for preterm birth and low birthweight may be maternal psychosocial stress during pregnancy. Exposure to chronic stress [2] and perceived stress [3–5] during pregnancy has been associated with preterm birth and low birthweight.

In a recent US study of 27 states, 70% of women reported experiencing at least one stressful life event in the year before giving birth [6]. Maternal stress in the form of stressful life events has been associated with preterm birth [2, 7–9] and low birthweight [5, 9, 10]. For example, a systematic literature review found women exposed to domestic violence during pregnancy to be at an increased risk for both delivering preterm and at low birthweight [11]. Further, a large Danish study found death or serious illness of close relatives early in pregnancy to be associated with an increased risk of preterm and very preterm birth [12], and pregnant women with active post-traumatic stress disorder (PTSD) were more likely to experience spontaneous preterm delivery compared to those without active PTSD [13].

The impact of individual stressors may be exacerbated in the presence of other stressors or abated by effective coping mechanisms or more social support. A prospective pregnancy cohort study in Canada observed a measure of cumulative psychosocial stress to be associated with an increased risk of late preterm birth, independent of other preterm birth risk factors [14]. When stratified by coping resources, a stronger association with higher levels of psychosocial stress was observed among women with lower perceived social support compared to those with higher levels of perceived support [14].

The current study explored stressful major life events and lack of social support (examples of data that can be efficiently collected) as potential risk factors for the outcomes of preterm birth and low birthweight employing data from controls in a large, population-based, U.S. case-control study of birth defects – The National Birth Defects Prevention Study. To explore whether different severities of preterm birth and low birthweight may have different etiologies, we used more specific sub phenotypes of these outcomes.

## Methods

Data for these analyses were from the National Birth Defects Prevention Study (NBDPS). The NBDPS is a population-based, multi-center, case-control study conducted at 10 sites located in Arkansas, California, Georgia, Iowa, Massachusetts, North Carolina, New Jersey, New York, Texas, and Utah. The NBDPS methods have been described in detail elsewhere [15]. Briefly, data were collected on cases with at least one of over 30 major birth defects and about 100 live-born, non-malformed controls who were randomly selected from each center each year from vital records (AR [2000–2011], GA [2001–2011], IA, MA, NC, NJ, UT) or birth hospitals (AR [1997–2000], CA, GA [1998–2001], NY, TX). The Institutional Review Boards of each center provided approval for the NBDPS and each participant provided verbal consent prior to interview. The telephone interview process did not proceed unless the woman consented to be interviewed. Our institution’s ethics board (Stanford University IRB) and the California Committee for the Protection of Human Subjects approved the use of the data analyzed in this paper.

In this analysis, we included only women delivering control infants due to the association between birth defects and preterm birth [16] and potential bias that may result from inclusion of women delivering case infants, and to be more representative of the general population of pregnant women. Participating women were administered a computer-assisted telephone interview in English or Spanish. Interviews took place between 6 weeks and 24 months after the expected date of delivery of the infant. Participation rates were 64% among eligible controls with a mean time from delivery to interview of nine months. For this analysis, we included women with an estimated date of delivery between January 2006 and December 2011, corresponding to an update of the questionnaire to include questions on stress and social support. The questions were derived from the Kaiser Permanente/California Department of Health Study of Pregnancy and Stress and parallel existing, validated stressful life events questionnaires [17–20]. Women were eligible for inclusion in this analysis if they had a singleton pregnancy and their infants were born between 20 and 42 weeks of gestation, had a birthweight between 500 and 6000 g, and had a birthweight within the acceptable range for the corresponding gestational week [21, 22]. There were 4678 eligible births. An additional 283 women were excluded because they were missing responses to the questions on stress, resulting in an analytic cohort of 4395 participants.

Outcomes of interest were preterm birth and low birthweight. Although preterm birth often accounts for many low birthweight infants, they were examined separately. Preterm birth was divided into “early preterm” (< 32 weeks gestation), “late preterm” (32–36 weeks gestation) and “term,” and low birthweight was divided into “very low birthweight” (< 1500 g), “low birthweight” (1500–2499 g) and “normal birthweight” (≥2500 g) [23]. Women were asked whether they experienced specific types of stress and support from three months before pregnancy through the third month of pregnancy (yes/no). Stress questions related to experiencing relationship difficulties, legal/financial problems, violence/crime, illness/injury, or a relative’s death. Additionally, women were asked if they had changed jobs or moved during the same time period. Support questions related to emotional support, financial help, and help with daily tasks. There was also one additional question about how often they had feelings of nervousness and stress measured on a 5-level Likert-type scale that was not included in later indices (see Appendix A for complete questions). Separate stress and support indices were created based on the cumulative number of questions for which the participant answered “yes.” The stress index ranged from 0 to 7 and the support index ranged from 0 to 3. We used the indices to create dichotomous variables for stress and social support. Participant responses skewed the stress index toward lower stress and the support index toward higher social support. Stress index values of 0–3 were considered “low stress”, while values of 4–7 were considered “high stress” after dichotomization at the median score. A support index score of 3 was considered “high support” and values of 0–2 were considered “low support” to create a large enough group for comparison. These variables were combined to form four categories (low stress/high support, high stress/high support, low stress/low support, and high stress/low support). Covariates were selected a priori based on their potential relationship to the outcomes of interest and to experiences of stress: maternal race/ethnicity, age, parity, education, pre-pregnancy body mass index (kg/m^2^), smoking, alcohol use, and intake of folic acid-containing vitamin/mineral supplements from the month before pregnancy through the first trimester of pregnancy.

Poisson regression with robust standard errors was used to calculate relative risks and 95% confidence intervals. Each stress and support question was analyzed individually with “no” as the reference group. The question regarding feelings of nervousness and stress was analyzed categorically with “sometimes” as the reference group since it tended to be the most frequent response. The stress and support indices were analyzed continuously and categorically with the stress reference group being “low stress” and the support reference group being “low support.” The combined index was analyzed categorically using low stress/high support as the reference group. Relative risks were calculated after adjustment for the previously selected covariates using complete case analysis.

Finally, a sensitivity analysis was performed excluding women who were taking benzodiazepines or selected antidepressants (based on those most frequently used in NBDPS [24, 25] to rule out potential differences in associations based on treatment.

## Results

Maternal characteristics by high or low cumulative stress scores are displayed in Table 1. A greater percentage of Black, non-Hispanic women, women younger than 25 years, smokers, and women who reported binge drinking were more likely to have a high stress score. Women over the age of 30, with more than a high school diploma, and who reported no smoking or drinking alcohol were more likely to have a low stress score.

**Table 1:** Participant Characteristics (% ^a^), National Birth Defects Prevention Study, 2006–2011 (*n* = 4395)

Characteristic	Low Stress Score (0–3)^**b**^	High Stress Score (4–7)^**b**^
	*N* = 4186	*N* = 209
**Race/ethnicity**		
White non-Hispanic	2351 (56.2)	96 (45.9)
Black non-Hispanic	404 (9.7)	43 (20.6)
Hispanic	1131 (27.0)	48 (23.0)
Other	299 (7.1)	22 (10.5)
Missing	1 (< 0.1)	–
**Age (years)**		
< 20	351 (8.4)	30 (14.4)
20–24	876 (20.9)	88 (42.1)
25–29	1247 (29.8)	45 (21.5)
30–34	1112 (26.6)	31 (14.8)
> 34	600 (14.3)	15 (7.2)
**Parity**		
Nulliparous	1656 (39.6)	89 (42.6)
Parous	2529 (60.4)	120 (57.4)
Missing	1 (< 0.1)	–
**Education**		
< High school	658 (15.7)	32 (15.3)
High school graduate	917 (21.9)	81 (38.8)
> High school	2602 (62.2)	96 (45.9)
Missing	9 (0.2)	–
**Smoking**^**c**^		
No	3559 (85.0)	108 (51.7)
Yes	625 (14.9)	101 (48.3)
Missing	2 (< 0.1)	–
**Use of folic acid-containing vitamin/mineral supplement**		
Began month before or 1st month	2423 (57.9)	86 (41.2)
Began 2nd - 3rd month	1277 (30.5)	79 (37.8)
Began later or none	447 (10.7)	41 (19.6)
Missing	39 (0.9)	3 (1.4)
**Alcohol use** ^**c**^		
None	2611 (62.4)	103 (49.3)
Some	1029 (24.6)	48 (23.0)
Binge drinking ^d^	511 (12.2)	57 (27.3)
Missing	35 (0.8)	1 (0.5)
**Pre-pregnancy body mass index (kg/m**^**2**^**)**		
Underweight (< 18.5)	200 (4.8)	10 (4.8)
Normal weight (18.5–24.9)	2041 (48.8)	91 (43.5)
Overweight (25.0–29.9)	925 (22.1)	48 (23.0)
Obese (> = 30.0)	849 (20.3)	55 (26.3)
Missing	171 (4.1)	5 (2.4)

^a^ Numbers may not add to 100% due to rounding

^b^ Indices reflect the number of questions that had a “yes” response

^c^ From one month before through three months after conception

^d^ Having four or more drinks on at least one occasion

Adjusted relative risks of early and late preterm birth are presented in Table 2. For women reporting stress from relationship difficulties, there was a suggestive increased risk of early preterm birth. There was also a suggestive increased risk for women reporting feelings of nervousness or stress somewhat or very often. There were no associations observed between questions regarding social support and early or late preterm birth or any of the stress/support combinations although two of the combinations were not calculated for early preterm birth due to small sample sizes.

**Table 2:** Association of Stressful Life Events and Social Support with Preterm Birth, NBDPS, 2006–2011

Exposures	Total (***N*** = 4134)	Early Preterm (***N*** = 38)	ARR^**a**^ (95% CI)	Late Preterm (***N*** = 266)	ARR^**a**^ (95% CI)
	No.	No. (row %)		No. (row %)	
**Stressful life events**^**b**^					
Relationship difficulties					
No	3432	27 (0.8)	Referent	211 (6.1)	Referent
Yes	702	11 (1.6)	1.9 (0.9,3.9)	55 (7.8)	1.2 (0.8,1.6)
Legal/financial problems					
No	3577	31 (0.9)	Referent	220 (6.2)	Referent
Yes	557	7 (1.3)	1.5 (0.6,3.4)	46 (8.3)	1.2 (0.9,1.7)
Violence/crime					
No	3843	36 (0.9)	Referent	237 (6.2)	Referent
Yes	291	2 (0.7)	NC	29 (10.0)	1.5 (1.0,2.2)
Illness/injury					
No	3550	32 (0.9)	Referent	224 (6.3)	Referent
Yes	584	6 (1.0)	1.1 (0.4,2.6)	42 (7.2)	1.2 (0.8,1.6)
Death of someone close					
No	3509	34 (1.0)	Referent	221 (6.3)	Referent
Yes	625	4 (0.6)	0.6 (0.2,1.6)	45 (7.2)	1.1 (0.8,1.5)
Changed jobs					
No	3111	29 (0.9)	Referent	199 (6.4)	Referent
Yes	1023	9 (0.9)	0.9 (0.4,1.9)	67 (6.5)	1.0 (0.7,1.3)
Moved					
No	3429	30 (0.9)	Referent	210 (6.1)	Referent
Yes	705	8 (1.1)	1.1 (0.5,2.5)	56 (7.9)	1.2 (0.9,1.7)
**Feelings of nervousness and stress**					
Never or almost never	1181	8 (0.7)	0.9 (0.4,2.3)	65 (5.5)	0.9 (0.6,1.2)
Sometimes	1728	13 (0.8)	Referent	108 (6.3)	Referent
Somewhat or very often	1225	17 (1.4)	2.0 (1.0,4.1)	93 (7.6)	1.2 (0.9,1.6)
**Stress index**^**c**^					
Low stress (0–3)	3934	36 (0.9)	Referent	246 (6.3)	Referent
High stress (4–7)	200	2 (1.0)	NC	20 (10.0)	1.4 (0.9,2.3)
**Social support**^**b**^					
Emotional support					
No	505	7 (1.4)	Referent	38 (7.5)	Referent
Yes	3629	31 (0.9)	0.7 (0.3,1.7)	228 (6.3)	1.0 (0.7,1.4)
Financial support					
No	601	7 (1.2)	Referent	42 (7.0)	Referent
Yes	3533	31 (0.9)	0.8 (0.3,2.0)	224 (6.3)	1.1 (0.8,1.5)
Help with daily tasks					
No	573	5 (0.9)	Referent	40 (7.0)	Referent
Yes	3561	33 (0.9)	1.2 (0.4,3.1)	226 (6.3)	1.0 (0.7,1.5)
**Social support index**^**c**^					
Low support (0–2)	984	13 (1.3)	Referent	71 (7.2)	Referent
High support (3)	3150	25 (0.8)	0.7 (0.3,1.4)	195 (6.2)	1.0 (0.7,1.3)
**Stress and social support combined**^**d**^					
Low stress, high support	3039	24 (0.8)	Referent	185 (6.1)	Referent
High stress, high support	111	1 (0.9)	NC	10 (9.0)	1.3 (0.7,2.5)
Low stress, low support	895	12 (1.3)	1.5 (0.7,3.2)	61 (6.8)	1.0 (0.7,1.3)
High stress, low support	89	1 (1.1)	NC	10 (11.2)	1.5 (0.8,3.0)

*Abbreviations*: *ARR* adjusted relative risk, *CI* confidence interval, *NC* not calculated

^a^ Relative risks were adjusted for maternal race/ethnicity, age, parity, education, prepregnancy body mass index (kg/m^2^), smoking, alcohol use, and intake of folic acid-containing supplements from the month before pregnancy through the first trimester of pregnancy

^b^ Complete questions can be found in Appendix A

^c^ Indices reflect the number of questions that had a “yes” response

^d^ Women were designated as having low stress if the stressful life events index score was 0–3 and as having high stress if it was 4–7. Women were designated as having low social support if the social support index score was 0–2 and as having high support if it was 3

Adjusted relative risks of very low birthweight and low birthweight are presented in Table 3. There was a suggestive increased risk of very low birthweight for women reporting stress from relationship difficulties, feeling nervous and stressed somewhat or very often. There was an increased risk of low birthweight for women who reported they or someone close to them were victims of abuse, violence, or a crime, and a suggestive increased risk for women reporting high stress and low social support. There were no associations observed between any support questions and low birthweight.

**Table 3:** Association of Stressful Life Events and Social Support with Low Birthweight, NBDPS, 2006–2011

Exposures	Total (***N*** = 4134)	Very Low Birthweight (***N*** = 31)	ARR^**a**^ (95% CI)	Low Birthweight (***N*** = 182)	ARR^**a**^ (95% CI)
	No.	No. (row %)		No. (row %)	
**Stressful life events**^**b**^					
Relationship difficulties					
No	3432	21 (0.6)	Referent	144 (4.2)	Referent
Yes	702	10 (1.4)	2.0 (0.9,4.4)	38 (5.4)	1.0 (0.7,1.5)
Legal/financial problems					
No	3577	25 (0.7)	Referent	152 (4.2)	Referent
Yes	557	6 (1.1)	1.6 (0.6,3.9)	30 (5.4)	1.2 (0.8,1.8)
Violence/crime					
No	3843	29 (0.8)	Referent	157 (4.1)	Referent
Yes	291	2 (0.7)	NC	25 (8.6)	1.8 (1.1,2.7)
Illness/injury					
No	3550	25 (0.7)	Referent	162 (4.6)	Referent
Yes	584	6 (1.0)	1.3 (0.5,3.3)	20 (3.4)	0.7 (0.5,1.2)
Death of someone close					
No	3509	29 (0.8)	Referent	153 (4.4)	Referent
Yes	625	2 (0.3)	NC	29 (4.6)	1.0 (0.7,1.5)
Changed jobs					
No	3111	24 (0.8)	Referent	136 (4.4)	Referent
Yes	1023	7 (0.7)	0.7 (0.3,1.7)	46 (4.5)	0.9 (0.6,1.3)
Moved					
No	3429	24 (0.7)	Referent	153 (4.5)	Referent
Yes	705	7 (1.0)	1.1 (0.5,2.7)	29 (4.1)	0.8 (0.5,1.2)
**Feelings of nervousness and stress**					
Never or almost never	1181	6 (0.5)	0.9 (0.3,2.5)	40 (3.4)	0.8 (0.5,1.2)
Sometimes	1728	10 (0.6)	Referent	74 (4.3)	Referent
Somewhat or very often	1225	15 (1.2)	2.2 (1.0,5.0)	68 (5.6)	1.3 (0.9,1.8)
**Stress index**^**c**^					
Low stress (0–3)	3934	29 (0.7)	Referent	168 (4.3)	Referent
High stress (4–7)	200	2 (1.0)	NC	14 (7.0)	1.3 (0.7,2.3)
**Social support**^**b**^					
Emotional support					
No	505	5 (1.0)	Referent	31 (6.1)	Referent
Yes	3629	26 (0.7)	0.8 (0.3,2.3)	151 (4.2)	0.8 (0.5,1.1)
Financial support					
No	601	3 (0.5)	Referent	29 (4.8)	Referent
Yes	3533	28 (0.8)	1.8 (0.5,6.1)	153 (4.3)	0.9 (0.6,1.4)
Help with daily tasks					
No	573	5 (0.9)	Referent	28 (4.9)	Referent
Yes	3561	26 (0.7)	0.9 (0.3,2.5)	154 (4.3)	1.0 (0.6,1.4)
**Social support index**^**c**^					
Low support (0–2)	984	8 (0.8)	Referent	53 (5.4)	Referent
High support (3)	3150	23 (0.7)	1.0 (0.4,2.4)	129 (4.1)	0.8 (0.6,1.1)
**Stress and social support combined**^**d**^					
Low stress, high support	3039	22 (0.7)	Referent	124 (4.1)	Referent
High stress, high support	111	1 (0.9)	NC	5 (4.5)	0.9 (0.4,2.2)
Low stress, low support	895	7 (0.8)	0.9 (0.4,2.4)	44 (4.9)	1.2 (0.8,1.7)
High stress, low support	89	1 (1.1)	NC	9 (10.1)	1.9 (1.0,3.9)

*Abbreviations*: *ARR* adjusted relative risk, *CI* confidence interval, *NC* not calculated

^a^ Relative risks were adjusted for maternal race/ethnicity, age, parity, education, prepregnancy body mass index (kg/m^2^), smoking, alcohol use, and intake of folic acid-containing supplements from the month before pregnancy through the first trimester of pregnancy

^b^ Complete questions can be found in Appendix A

^c^ Indices reflect the number of questions that had a “yes” response

^d^ Women were designated as having low stress if the stressful life events index score was 0–3 and as having high stress if it was 4–7. Women were designated as having low social support if the social support index score was 0–2 and as having high support if it was 3

## Discussion

Overall, we did not observe individual stressful events to be moderate-to-strong risk factors for shortened gestation or having a low birthweight infant except for an increased risk of having a low birthweight infant if the woman or someone close to her was a victim of violence or crime. There were no significant associations observed between responses to social support questions and these adverse birth outcomes.

Stress could affect fetal development through a variety of mechanisms, including impacts on thrombotic and inflammatory pathways, hypoxia, oxidative stress, placental development and uterine contractility [26–28]. Stress could also affect fetal development through the neuroendocrine pathway. Measures of psychosocial stress and biological effectors of stress have been shown to predict levels of placental corticotropin-releasing-hormone and increases have been associated with spontaneous preterm labor [28, 29]. It is possible that these mechanisms reflect chronic stress mechanisms versus more acute stress. Additionally, it is possible that unobserved fetal loss may bias our risk estimates given that psychosocial stress has been associated with miscarriage in some studies [30]. To attempt to assess this hypothesis, we performed an ad hoc effect modification analysis to estimate if risk of preterm birth is modified by infant sex. It has been posited that male fetuses may be more likely to be “culled,” i.e. before they can be observed to occur as a fetal death or live birth, in response to stressors [31, 32]. We observed higher risks of preterm birth for female infants among women experiencing relationship difficulties, job changes, and measuring high (≥4 “yes” responses) on the stress index (results not shown). We also observed higher risks for female infants born to women experiencing high stress/low support (ARR 3.0 vs. 0.67; p-heterogeneity = 0.05). These results support this hypothesis, which could explain some of the null findings if the pregnancies with male fetuses were more likely to result in fetal loss in response to psychosocial stress. However, infant sex differences may also be due to differences in reactions to stressors, inflammation, or other factors.

The associations observed between women who reported they or someone close to them were victims of abuse, violence, or a crime and low birthweight and the suggestive associations between relationship difficulties and very low birthweight and preterm birth support results from the literature. Previous studies have reported associations between abuse and adverse pregnancy outcomes, with both physical and psychological mechanisms proposed [11, 33]. Exposure to violence or crime and relationship issues may both encompass a level of domestic or other abuse although our questionnaire did not elicit this level of specificity. A previous study of more specific major life events also observed both negative and positive associations with birthweight, depending on the stressful event [5]. While our results were not as heterogeneous, it is possible that different events lend themselves to different levels or chronicity of stress.

Previous studies of maternal social support and low birthweight have had conflicting results [34, 35]. We observed a suggestive association between high stress/low support and having a low birthweight infant but not preterm birth as seen in a previous study [14]. However, we were unable to calculate ARR estimates for a few of the combinations of stress and social support for the more severe adverse outcomes due to sample size. Additionally, we were also unable to calculate specific combinations of stress and social support (i.e., specific life events and types of support) due to sample size and cannot rule out social support being a buffer of certain stressful life events and not others. McDonald et al. utilized data from more detailed questionnaires to measure stress and social support and thus may have been better able to discriminate between those with high and low levels of social support [14]. Many of our participants also responded yes to all of the social support questions, limiting comparisons between low and high social support.

Medication for stress-related disorders such as anxiety and depression have been associated with preterm birth and low birthweight in some studies. In one review, antidepressant use later in gestation was associated with preterm birth, independent of depression [36]. In another study, benzodiazepine use was associated with low birthweight and both benzodiazepine use, and serotonin reuptake inhibitor use appeared to be associated with shortened gestation [37]. To assess potential effects of medication use on any observed associations, we performed a sensitivity analysis excluding 266 women who were taking any of the commonly used benzodiazepines (alprazolam, clonazepam, diazepam, and lorazepam) or selective-serotonin reuptake inhibitors (sertraline, paroxetine, fluoxetine, escitalopram, or citalopram), or bupropion. However, we did not observe a substantial change in risk after excluding women reporting use of these medications (data not shown).

One limitation of this study is that the interview asks about the presence or absence of selected stressors without taking into account duration or chronic nature of the stressor and thus may be a better measure of acute versus chronic stress. It is possible that some of the women responding yes to certain questions did not experience a level of stress of sufficient intensity to impact pregnancy outcomes. The questions also ask if the woman or someone close to her experienced some of the stressful situations. It is possible that stress in response to the situation of another person may impact a pregnancy in different ways than direct stress to oneself. The questions also asked for a participant’s experience during a specific point in gestation from pre to early pregnancy. One study observed that only perceived stress during the second trimester was associated with preterm birth [38], which we would not be able to estimate in this study. Based on the nature of the retrospective data collection, recall is also a potential issue as well as social desirability bias, or an unwillingness to report certain negative experiences. However, this study focused on major life events which helped to limit potential biases. Additionally, small sample size for some outcome groups and low prevalence of stressful life events hindered calculation of certain risks.

Strengths of this study include the population-based sample and standardized interview examining multiple possible situations of stress and social support. The questionnaire examined these situations utilizing major life events that are relatively common and easy to recall among women who are representative of the general population. While we did not observe specific associations for many of the separate stress or support questions, we gained some insight regarding the potential association with cumulative stressors. The indices were created based on the assumption that effects of stressful and supportive life events are additive [39], and we did observe some positive associations with a greater number of stressful events. It is possible that our results point to a greater stress load rather than specific stressful events as related to adverse birth outcomes. We also examined adverse birth outcomes more granularly than simply low birthweight or preterm birth. Although sample size was limited for early preterm birth and very low birthweight, the differences in results between the different subgroups of low birthweight and preterm birth add support to the hypothesis that differences by severity are due to different etiologies. Preterm births were likely the cause of some of the low birth weights in this population. We considered low birthweight instead of small for gestational age in order to better match outcomes used in other studies, allowing our data to better contribute to the synthesis of data across studies. Our sample was also randomly sampled from each study centers’ catchment area and thus should be generalizable to these 10 areas throughout the US, though not the entire US.

Given that exposure to stress is relatively common, and that social support may not be adequate to attenuate potential harm due to stress, it is important to continue to understand the effects of various types of stress, both acute and chronic, in different populations and what methods of support or treatment may buffer its negative effects. It is important to keep developing ways to improve mental wellbeing, particularly during pregnancy.

## Conclusions

Our results add some support to prior evidence that certain stressors, like being or knowing a victim of violence or a crime, may be associated with an increased risk of selected adverse birth outcomes. We did not find strong evidence that social support buffered the observed risks in our study but given the high levels of perceived social support in our population, we may not have had an adequate distribution to fully investigate its potential effects.

## Supplementary information

**Additional file 1.** Questions Related to Stressful Life Events and Social Support in the National Birth Defects Prevention Study from 3 months before pregnancy through the 3rd month of pregnancy.

## Data Availability

The datasets generated and/or analysed during the current study are not publicly available to guarantee the confidentiality of participants and to ensure that data are used in accordance with their consented purposes but are available from the corresponding author on reasonable request. https://www.cdc.gov/ncbddd/birthdefects/nbdps-public-access-procedures.html

## Abbreviations

PTSD: Post-traumatic stress disorder
NBDPS: National Birth Defects Prevention Study
RR: Relative risk
CI: Confidence interval
