# Supplementary material for: Periconceptional stressors and social support and risk for adverse birth outcomes
Source: BMC Pregnancy Childbirth. 2020 Aug 24;20:487. doi: 10.1186/s12884-020-03182-6 (PMC7446063; doi:10.1186/s12884-020-03182-6)
Supplement: Supplementary file 1 — Additional file 1. Questions Related to Stressful Life Events and Social Support in the National Birth Defects Prevention Study from 3 months before pregnancy through the 3rd month of pregnancy. [file 12884_2020_3182_MOESM1_ESM.docx]

Appendix A.

Questions Related to Stressful Life Events and Social Support in the National Birth Defects Prevention Study from 3 months before pregnancy through the 3^rd^ month of pregnancy

Stressful Life Events:

1. Did you experience any serious relationship difficulties with your husband or partner or become separated or divorced?

2. Did you or your husband or partner have any serious legal or financial problems?

3. Were you or someone close to you a victim of abuse, violence, or crime? Remember you just have to indicate yes or no.

4. Did you or someone close to you have a serious illness or injury?

5. Did someone close to you die?

Social Support:

6. Could you count on anyone to provide you with emotional support, such as talking over a problem or helping with a difficult decision, if you had needed it?

7. Could you count on anyone to provide you with help financially, such as paying bills or providing food or clothes, if you had needed it?

8. Could you count on anyone to provide you with help with daily tasks, such as grocery shopping, child care, or cooking, if you had needed it?

Additional:

9. How often did you feel nervous and stressed? (Never, Almost Never, Sometimes, Somewhat Often, Very Often)

Note: A series of questions about occupational history and residential history were asked in separate sections of the questionnaire. From these questions, we determined whether mothers had moved or changed jobs during the periconceptional period.
